# Isolation and Characterization of Beneficial Bacteria from Food Process Wastes

**DOI:** 10.3390/microorganisms9061156

**Published:** 2021-05-27

**Authors:** A-Leum Kim, Seunghye Park, Yoon-Kyoung Hong, Ji-Hwan Shin, Se-Hwan Joo

**Affiliations:** R&D Center, Cosmicgreen Ltd., Seoul 08390, Korea; lucia-kim@cosmicgreen.co.kr (A.-L.K.); shpark@cosmicgreen.co.kr (S.P.); ykhong@cosmicgreen.co.kr (Y.-K.H.); wiseforests@cosmicgreen.co.kr (J.-H.S.)

**Keywords:** beneficial microorganisms, plant growth regulator, extracellular lysis

## Abstract

Significant quantities of food waste are accumulated globally on an annual basis, with approximately one-third of the food produced (equivalent to 1.3 billion tons of food) being wasted each year. A potential food waste recycling application is its utilization as a soil conditioner or fertilizer, whereby it increases the soil organic content and microbial biomass. This study evaluated the effectiveness of food waste as a microbial resource by analyzing the microbial community composition and isolating plant growth-promoting bacteria (PGPB) in food waste obtained from various sources. High-throughput sequencing identified 393 bacterial operational taxonomic units in the food process waste (FPW) samples. Moreover, the results showed that Firmicutes was abundant in the waste samples, followed by Bacteroidetes and Proteobacteria. A total of 92 bacteria were isolated from FPW. Moreover, the cultivable strains isolated from FPW belonged to the genus Bacillus, followed by Streptomyces and Proteus. Six isolated bacteria exhibited beneficial traits, including indole acetic acid production, antifungal resistance and extracellular lysis. FPW is a valuable microbial resource for isolation of PGPB, and its use as a fertilizer may enable a reduction in chemical fertilizer usage, thereby mitigating the corresponding adverse environmental impacts on sustainable crop development.

## 1. Introduction

As the demand for food is resource-dependent, significant quantities of food loss and waste accumulation are generated globally on an annual basis, with approximately one-third of the food produced (i.e., 1.3 billion tons of food) wasted every year [1]. Moreover, private households are the most significant contributors to food waste [2].

Various management practices exist for handling solid waste from food production, and because these wastes have high nutrient levels and water content, and may encourage bacterial growth and fermentation, odors and other environmental problems may arise [3,4]. The majority of food waste is transported for disposal and incineration to landfill sites, leading to additional costs and environmental pollution [5]. However, some waste may be dried and formed into animal feed, owing to its calorific value [6]. Food waste can also be utilized as a soil conditioner or fertilizer [7]. This can be achieved by distributing untreated food waste on soil surfaces, thereby increasing the soil organic content and microbial biomass [8].

Food waste is often used to produce compost. However, the nutrition contained in compost is consumed during microbial growth [9]. Therefore, organic food waste composting is only possible via the addition of a function-specific microbial community [10].

Microbial communities consist of various microbial species, ranging from cultivable and non-cultivable strains, which can interact with each other in the environment in which they co-exist [11]. During food waste composting, these microorganisms degrade food waste into smaller particles before composting [10]. Moreover, the microbial community composition is an essential determinant of the compost consistency, as it changes and develops during the composting process [12].

Beneficial microbes produce enzymes that degrade organic matter and mitigate the effects of harmful pathogens [13,14,15]. Furthermore, the enzyme-producing capabilities of bacteria are an important research topic, owing to the efficiency of these enzymes in catalyzing chemical reactions in living organisms [16]. More specifically, bacteria-produced enzymes break down organic material into a stable material through various biochemical processes; thus, they are essential tools for waste conversion [10]. Moreover, food waste consists predominately of carbohydrates, proteins, and fats. Therefore, the enzymes produced by bacteria can break the bonds between macromolecules and are beneficial in the waste conversion process [17].

Despite the importance of bacteria-produced enzymes in the waste conversion process, relatively little is known about the relationship between food waste and the nature of microbial species of different origins and processes. Therefore, this study aimed to isolate, screen, and characterize food-waste borne bacteria which affect plant growth and other bioconversion processes. These beneficial microorganisms may be valuable as compost-fortifying agents that can suppress plant pathogens and stimulate plant development.

## 2. Materials and Methods

### 2.1. Sample Collection

The food process waste (FPW) samples, including used rice grounds (FPWR), vegetable by-product (FPWV), and compost (FPWC) used for analysis, were obtained from several food companies. Composting of samples was conducted at our research compost facility. For microbiological assessments, each sample was placed in a sterile zip-lock plastic bag to maintain aseptic conditions and stored at 4 °C prior to analysis.

### 2.2. Culture Conditions and Bacterial Isolation

Bacterial isolation from the FPW samples was performed using standard methods and serial dilution plating. The sample suspension was prepared by adding food waste (1 g) to 10 mL of sterile water and shaking vigorously for ≥120 min. The diluted sample was then allowed to settle for a short period. The bacterial stock was serially diluted 10-fold over a range of 10^−1^ to 10^−6^ and plated (0.1 mL per plate) in triplicate. The bacteria were incubated on solid media containing the yeast extract mannitol (YEM) in an ambient CO_2_ atmosphere at 30 °C for several days. A single isolated colony was then streaked on the same agar plate and incubated at 30 °C for 24 h. Isolated strains were cultured and maintained in a Luria–Bertani (LB) medium. All classified strains were stored in a 25% glycerol medium at –80 °C, creating a culture collection of bacteria from various environments.

### 2.3. Microbiome Analysis

After resuspending all the samples, the suspensions were bead-beaten and centrifuged at 14,000× *g* for 10 min. The supernatants were fused with nuclease-free water, and polymerase chain reaction (PCR) amplification was performed. DNA amplification targeted the V3-V4 regions of the bacterial 16S rRNA gene using 341F and 805R primers. PCR products were sequenced using an Illumina MiSeq sequencing system (Illumina, San Diego, CA, USA) at ChunLab. Classification and identification of the 16S rRNA gene sequences for phylogenetic analysis were processed in EzBioCloud using the microbiome taxonomic profiling pipeline (ChunLab, Inc., Seoul, Korea) [18].

### 2.4. Taxonomical Assignation

For isolation and identification of primarily isolated microorganisms by morphological screening, genomic DNA was extracted and amplified using the universal primers 27F (5′-AGAGTTTGATCCTGGCTCAG-3′) and 1492R (5′-GGTTACCTTGTTACGACTT-3′). The polymerase chain reaction was conducted under the following conditions: 95 °C for 5 min, 30 cycles of 95 °C for 30 s, 52 °C for 30 s, 72 °C for 90 s, and a final extension at 72 °C for 10 min. Amplified PCR products were purified using a PCR purification kit (Geneall Co., Songpa-gu, Seoul, Korea) and sequenced (Cosmogenetech, Seongdong-gu, Seoul, Korea). Taxonomy was defined using the NCBI GenBank database (https://blast.ncbi.nlm.nih.gov; accessed on 14 July 2021). Sequence data were assembled and analyzed using FinchTV (Geospiza, Inc., Seattle, WA, USA).

### 2.5. Phylogenetic Analysis

The 16S rRNA gene sequences of the type strains and other strains closely related to the 44 isolated strains were obtained from the NCBI database to construct a phylogenetic tree. The Clustal_X program was used to perform multiple sequence alignments [19]. Phylogenetic trees were constructed by the maximum-likelihood method based on the Jukes–Cantor model [20] using MEGA-X (ver. 10.2.4). All positions containing gaps and missing data were eliminated. The final dataset contained 800-base pair (bp) positions in total.

### 2.6. In Vitro Identification of Indole-3-Acetic Acid (IAA)

The bacterial culture supernatant was evaluated using the colorimetric method to classify auxin-producing isolates. The isolates were incubated in the LB broth supplemented with 0.1% L-tryptophan as a precursor (IAA). After the incubation period, Salkowski’s reagent [21] was mixed with each of the culture supernatants in a 2:1 ratio. If IAA or similar compounds were present in the supernatant, they developed a pink color upon reaction with the Salkowski reagent, which was then compared to a standard curve of indole-3-acetic acid (Sigma-Aldrich, Saint Louis, MO, USA) ranging from 10–100 μg/mL obtained using a spectrophotometer (Spectrophotometer UV5, Mettler Toledo, Columbus, OH, USA) at 530 nm.

To obtain high-resolution spectra of IAA, we used a high-resolution approach applied to a liquid chromatograph with tandem mass spectrometry (LC/MS–MS; Ultimate 3000 RS-Q-Exactive Orbitrap Plus, Thermo Scientific, Waltham, MA, USA) in positive ionization mode. Chromatographic separation was performed on a Column Aquity UPLC BEH C18 (100 mm × 2.1 mm, 1.7 μm); mobile phase A (0.1% formic acid in water), B (0.1% formic acid in acetonitrile), gradient elution program: 0–1.5 min (5% B), 1.5–10 min (100% B), 10–13.5 min (100% B), and 13.5–14 min (5% B). The flow rate was set to 0.4 mL/min and the injection volume was 5 µL. All screening data were acquired using full MS scan/dd-MS2 mode. The full scan spectra were acquired across the range of *m/z* 80–1000. The mass spectrometer was calibrated using an automated, calibrated delivery system. IAA was identified and qualified by comparing the retention time and prominent ions with those of the standard IAA.

### 2.7. Detection of Extracellular Enzyme Activity

The isolated bacterial strains were screened to produce four enzymes: protease, cellulase, lipase, and ligninolytic enzymes. Each bacterial strain was separately streaked on the respective substrates, namely, skim milk [22], carboxymethyl cellulose (CMC) [23], Tween 80 [24], and Azure B [25], which had been amended to agar plates. Petri plates were incubated overnight at the appropriate temperature. The plates were then treated with an indicator solution, and the development of a transparent zone around bacterial colonies was considered to indicate enzyme activity.

### 2.8. Antifungal Assay

All isolated bacteria were tested for antifungal activity against three pathogens: Phytophthora blight (*Phytophthora capsici*, KACC 40158), sheath blight (*Rhizoctonia solani* AG-1, KACC 40101), and gray mold (*Botrytis cinerea*, KACC 40574). A fungal plug was placed at the center of the PDA plate and each isolated bacterium was inoculated 2.5 cm from the center. The plates were incubated at 30 °C for 24 h. Strains with the capacity to inhibit the proliferation of the fungus were selected and preserved.

## 3. Results

### 3.1. Bacteria Diversity and Richness

A mean number of reads per sample of 62,389 was obtained from our analysis, and the results for each sample are summarized in Table 1. The CL community program (Chunlab Inc., Seoul, Republic of Korea) was used for the taxonomic assignment of each pyrosequencing read. Based on the sample size, Good’s coverage valuewas 0.99, indicating that the sample size selected in this study was sufficient to accommodate all bacterial diversity (Table 1).

The Shannon diversity index ranged from 1.185–5.287 for the three samples, and the Simpson index ranged from 0.022–0.414. The FPWR Chao1 was 101.1, and the FPWV and FPWC were 377.5 and 2398.5, respectively. The bacterial diversity for FPWR was significantly lower than that for FPWC, based on a comparison of the diversity indices (Shannon and Chao1).

**Figure 1:**
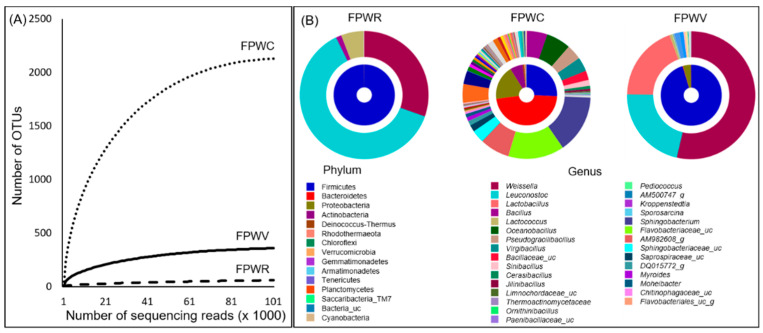
Rarefaction curve and bacterial distribution in FPW samples (**A**) The rarefaction curve of observed species which were evaluated by 16S sequences. (**B**) Double pie charts of bacterial communities in FPW samples. The inner and the outer circle indicate the phylum and the genus compositions of the bacterial communities.

Rarefaction curves that plot the number of operational taxonomic units (OTUs) against the number of sequence reads for the three samples are shown in Figure 1A. The rarefaction study was consistent with the diversity indices. All the indices indicated that FPWR and compost had the lowest bacterial diversity. Similarly, rarefaction analysis showed that the OTUs per valid sequence read were lower in the FPWR samples than in the FPWV and FPWC samples.

Our analysis revealed a total of 15 phyla, 396 genera, and 765 species. There were 50, 256, and 393 species-level OTUs for FPWR, FPWV, and FPWC, respectively. The average bacterial composition was obtained via integration of the clustering results, in addition to the abundance of each sample by phylum. Firmicute were most frequently detected, followed by Bacteroidetes and Proteobacteria. The species composition at the phylum level was different in FPWC when compared to other samples. At the genus level, when the top 30 genera were compared between different samples, the genera composition of FPWC differed from others FPW samples (Figure 1B).

### 3.2. Isolation of Beneficial Bacteria from FPW Samples

Various selective media were used for culturing bacterial populations from the three samples using quadrant streaking for four cycles. The number of cultivable bacteria in the FPW ranged from 2.0 × 10^6^ to 4.2 × 10^6^. The initial count was up to 28 for the YEM medium, which eventually decreased to 22. The pattern of colony reduction was also observed in LB media, in which 81 were initially created and were subsequently reduced to 70 colonies. After consecutive selection, 92 distinguishable isolates were collected at the end of the selection process. All isolates underwent morphological and biochemical testing.

### 3.3. Biochemical Characteristic of Isolated Bacteria

**Figure 2:**
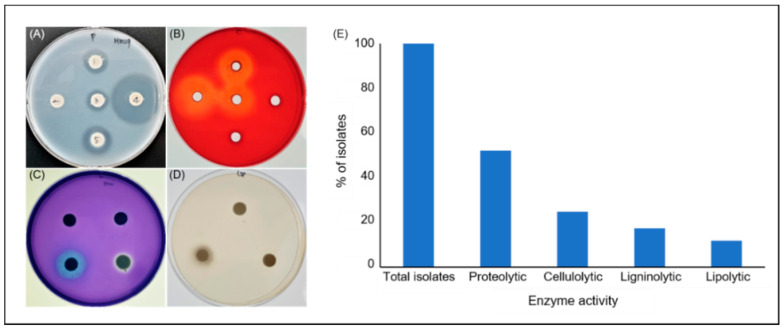
Production of extracellular lytic enzymes by the bacteria from FPWs. Plate assay showing (**A**) proteolytic, (**B**) cellulolytic, (**C**) ligninolytic, and (**D**) lipolytic enzyme activities of the isolated bacteria. The assay was performed by inoculation of the bacterial isolates on agar media containing suitable substrates specific for each enzyme activity. After incubation, media were treated with specific staining solutions to visualize the formation of clear halos around bacterial colonies, indicating production of the respective enzymes (representative plates showed). (**E**) Distribution (%) of extracellular hydrolytic enzyme activities among the isolates.

All isolates were grown on plates filled with media used for the detection of enzyme activities. The overall extracellular enzyme activities of the tested isolates are presented in Figure 2.

#### 3.3.1. Proteolytic Assay

To select effective isolates, the diameters of the transparent zones formed by the isolates (Figure 2A–D) were measured. The diameters of transparent zones produced by the proteolytic bacteria ranged from 3–11 mm. A total of 48 isolates with the ability to produce a transparent zone in the skim milk medium were identified. Among the isolates, 16 showed a high proteolytic activity.

#### 3.3.2. Cellulolytic Assay

A total of 23 isolates were selected using CMC-Na solid media followed by Congo red staining. The diameter of the transparent zones in the medium ranged from 3–11 mm. Among all the isolates, the enzyme activity of 16 was higher than that of the other strains, indicating that this strain exhibits stable inheritance and a strong cellulolytic activity.

#### 3.3.3. Ligninolytic Assay

Sixteen lignin-degrading bacteria were isolated, and Azure B was added directly to the agar as a carbon source. The bacterial colonies were visible within average incubation times of 16–24 h. The results confirmed that Azure B was bleached because of the lignin-degrading activity of enzymes released by the microorganisms.

#### 3.3.4. Lipase Assay

Lipase production was observed in the medium containing Tween-80, producing transparent zones around the colonies. Moreover, a visual observation of the size of the intensification zone further indicated lipolytic activity. Of the 92 strains tested, only 11 strains were selected.

### 3.4. Antifungal Activity Assay

Numerous bacteria produce antifungal compounds [26]. In the present study, 13 isolates exhibited antifungal activity against pathogens that frequently occur in crops and affect production [27]. The results are listed in Table 2. Moreover, measuring the growth inhibition zone through the replacement culture in an antibacterial medium revealed that all the strains had at least one inhibitory activity against the following three pathogens: *P. capsici, R. solani,* and *B. cinerea.*

### 3.5. IAA Assay and Determination

**Figure 3:**
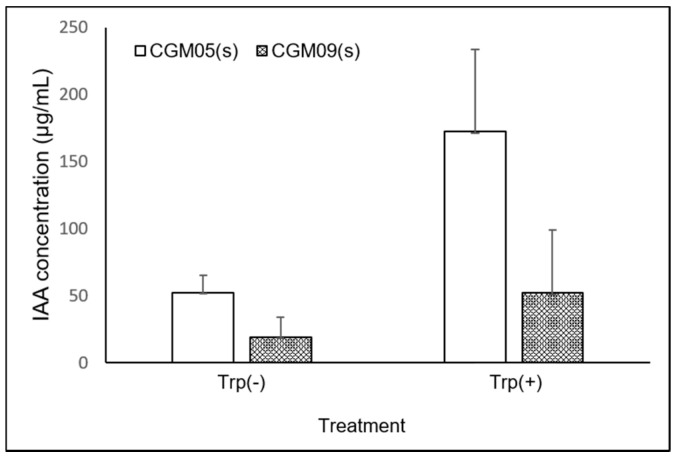
Comparison of IAA production by the bacterial isolate, CGM05, and CGM09, in LB medium with or without Trp. Mean of three independent experiments.

Synthesis of the IAA plant hormone observed in the media with or without supplementation with tryptophan (Trp). The presence of IAA was indicated by a shift in the color of the medium to pink or red. The amount produced was quantified for the 13 strains showing auxin-generating activity, and the range of the auxin yield of the tested bacteria was approximately 8.2–52.0 μg/mL in Trp-free medium (Figure 3). Moreover, high IAA concentrations were detected in the CGM05 and CGM09 strains (172.3 and 52.0 μg/mL, respectively). In the Trp-free medium, the amounts of IAA released by CGM05 and CGM09 were 30% and 13%, respectively, of the amounts released when Trp was used.

**Figure 4:**
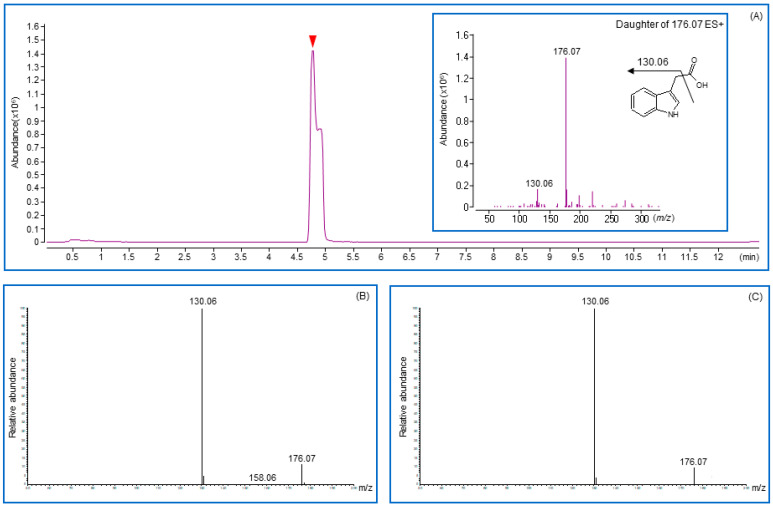
Chromatograms and fragmentation patterns were obtained for indole-3-acetic acid by using liquid chromatography with tandem mass spectrometry (LC/MS-MS). (**A**) Total Ion Chromatograms and fragmentation pattern (inbox) for IAA standard. (**B**,**C**) Chromatograms and fragmentation patterns were obtained from CGM05 and CGM09 strain cultured media. Solid arrowhead in chromatograms indicates the retention time for indole-3-acetic acid.

The auxin analog produced by the isolate was detected in the supernatant of the culture and bacterial cell wall components. A strong signal was detected in the culture supernatant. However, a negligible indication was observed in the bacterial cell wall components, indicating the ability of the strain to secrete IAA analogs out of the bacterial cells. The structure of the compound secreted by the *Providencia* and *Proteus* strains was confirmed by comparison with standard IAA and indole butyric acid (IBA), exhibiting identical retention times, mass spectra, and MS–MS spectra under the same conditions. IAA showed a mass spectrum with a positive ion ([M + H]+) peak at *m/z* 176, and the fragment ion peak at *m/z* 130 of the culture supernatant sample, showing the same pattern as the standard IAA compound (Figure 4). Therefore, the peaks at 4.7 min for the CGM05 and CGM09 supernatants of the culture sample were identified as IAA.

### 3.6. Identification of Bacteria

**Figure 5:**
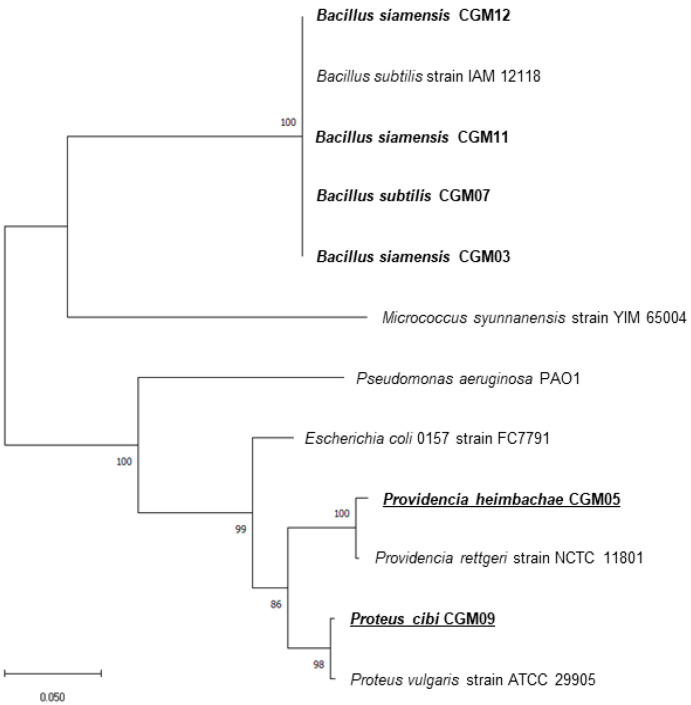
Phylogenetic positions of the isolates and their close relatives. The tree was constructed by using the neighbor-joining method. Scale bar indicates 0.05 substitutions per nucleotide position.

Isolates were screened for colony morphology, and extracellular and antifungal activities were further identified using 16S rRNA sequencing. Six identified isolates belonged to the genera *Bacillus*, *Proteus*, and *Providencia*. Sample CGM07 was related to *Bacillus subtilis* (100% similarity); CGM03, CGM11, and CGM12 were significantly related to *Bacillus siamensis* (100%); CGM09 was related to *Proteus cibi* (100%); and CGM05 closely resembled *Providencia heimbachae* (99.87%). The phylogenetic tree was developed using MEGA-X (ver.10.2.4) (Figure 5).

## 4. Discussion

In this study, we determined that beneficial microorganisms such as plant growth-promoting bacteria (PGPB) were present in various FPWs obtained from recyclable materials, indicating that unused resources provide valuable microbial resources. Various raw materials have been used for composting to promote the growth of various types of microorganisms. Food waste is particularly rich in fiber, nitrogen, potassium, and other nutrients. Food waste degradation processes occur with the aid of naturally occurring microorganisms, of which diverse types have been identified. These microorganisms can also contribute to plant growth and soil fertility. Beneficial assays include direct and indirect measurements of microbial activity that can promote plant growth, such as phytohormones, extracellular lytic enzymes, and antifungal activity [28].

Among the 92 bacteria isolated in the present study, 55 showed extracellular lytic enzyme activities and 13 showed antifungal activity through diffusible substances in agar. Moreover, five isolates showed potential as biological control agents (Table 2). These bacteria may exert their antifungal activity by producing extracellular lytic enzymes, siderophores, salicylic acid, antibiotics, and volatile metabolites such as hydrogen cyanide [29,30,31,32,33,34]. The resulting bacteria can be used to design new environmentally friendly methods to control plant pathogens (particularly *P. capsici*, *R. solani*, and *B. cinerea*) without using chemical fungicides that contaminate agricultural soils.

Bacteria produce metabolites that interact with plants in various ways [35]. Phytohormones (auxins, cytokinins, gibberellins, abscisic acid, and ethylene) can be synthesized by bacteria and play roles in stimulating plant growth [36]. Of these, auxins play a crucial role in rooting, apical domination, and budding [37]. Auxins refer to various chemically different compounds, most commonly IAA, IBA, and indol-3-propionic acid (IPA). Several related chemicals (e.g., indole-3-acetonitrile, indole-3-acetamide, and indole-3-acetaldehyde) serve as biosynthetic intermediates and are not known to have hormonal effects [38]. IBA and IPA enhance plant growth and are categorized as potent auxins [39]. In this study, CGM09 showed higher auxin production than CGM05, and Salkowski’s color change test indicated that the CGM09 culture media contained different auxin molecules compared to those present in CGM05. Moreover, chromatographic analysis using LC–MS showed that CGM09 produced a small amount of IAA and putative IPA as potent auxins (unpublished data). The IAA-producing potential is an important attribute of PGPB; CGM05 (*P. heimbachae*) exhibited the highest auxin activity among the 92 isolates tested, and CGM09 (*P. cibi*) was a potential auxin producer.

Trp is the primary precursor of IAA biosynthesis in microbes [40,41]. Two separate pathways, namely, Trp-dependent and Trp-independent pathways, broadly characterize IAA biosynthesis based on the distinct intermediates involved in IAA biosynthesis in bacteria [42,43]. Although Trp-independence is perceived to occur in bacteria [44,45], no specific enzymes in this pathway have been characterized. Moreover, the bacterial strains were also able to produce trace amounts of IAA in the absence of Trp, and larger amounts in the presence of Trp. The amount of IAA released in the presence of Trp exceeds the amount released when Trp is not supplemented [38,43].

CGM05 and CGM09 were able to produce IAA in the absence of an exogenous Trp supply. We assumed that bacteria may have the capacity to synthesize Trp as an endogenous precursor or that the Trp-independent IAA biosynthesis mechanism may operate in these two strains. However, the Trp-independent pathway has not been comprehensively characterized in bacteria, and no related enzymes have been identified in this pathway [44]. Thus, the existence of Trp-independent pathways has not been verified and further research is required.

Several isolates in this study may have produced antifungal compounds or enzymatic activities. It can be assumed that the presence of bacteria promotes plant growth by participating in defense-related plant reactions and inhibiting mycelium growth and spore germination [46]. The use of *Bacillus* sp. strains as biological control agents has been reported for harmful plant pathogenic bacteria, including *B. subtilis*, *B. thuringiensis*, *B. licheniformis*, and *B. amyloliquefaciens* [47]. In particular, studies on the antibacterial activity of B. subtilis have been conducted [47,48,49,50]. Members of the *Bacillus* genus produce various antifungal compounds, many of which are identified as peptides, lipopeptides, and phenolic derivatives [51,52]. Research to identify novel secondary metabolites with diverse biological activities in various environments has gained significant attention [53,54]. Despite this, relatively little research has focused on the applications of biological activities in agriculture [53]. Therefore, this study conducted pioneering research on bacterial applications to crop growth, and the five selected isolates showed excellent antifungal activity and, hence, can be used as biological control agents for suppressing harmful pathogens in crops.

Several composting investigations have also established the effect of microbial inoculation on compost consistency [55]. Typically, accelerating the operation time of the degradation process produces high-quality compost, owing to microbial action (including CMCase, xylanase, and lignin peroxidase) and organic matter degradation [56,57,58]. In addition, the increased enzymatic behavior and shortened initial lag time of the biological process compensate for the successful microbial acceleration of composting [59]. Moreover, microbial inoculation can effectively remove odor emissions (mostly volatile organic compounds) and produce compost with a higher nutritional value [60,61].

In this study, six beneficial isolates with auxin biosynthetic activity, with or without multiple extracellular lytic and antifungal activities, were identified. This advances our understanding of the regulatory system underlying soil remediation and the plant growth-promoting effects of this bacterium. Since microorganisms play a major role in food waste processing as well as composting, knowledge of the behavior and dynamics of microbial communities is necessary for process optimization [62]. This is because the presence of different bacteria can positively or negatively affect composting, and modification of the type and amount of input materials can alter the microbial community [63].

Considering these results, the selection of multifunctional strains is essential. Because of the positive effect of the inoculants on plant growth, optimizing their application for maximum impact on vegetable crops is critical. Utilizing various types of FPW reduces the need for chemical fertilizers for commercial crops, reducing the effect on crop growth, soil properties, and the environment. Further research should aim to determine the effects of potential food-waste fertilizers on plant growth and the soil environment.

## Figures and Tables

**Table 1:** Comparison of diversity of microorganisms isolated from different food process wastes (FPWs); used rice grounds (FPWR); vegetable by-product (FPWV); and compost (FPWC).

Sample	Reads		Observed Species (OTUs) ^a^	Alpha Diversity			Coverage
	Raw	Valid		Shannon	Simpson	Chao1	
FPWR	31,012	30,155	50	1.185	0.414	101.1	0.99
FPWV	107,597	86,350	256	1.930	0.313	377.5	0.99
FPWC	87,873	70,664	393	5.287	0.022	2398.5	0.99

^a^ Isolates were assigned to operational taxonomic units (OTUs) with a cutoff of 97% identity of the basis of their partial 16S rRNA gene sequences.

**Table 2:** Distribution of the morphological, characteristics, extracellular enzyme, antifungal, and auxin-producing activities among the isolates.

Isolated Strains	Organic Molecule Degradation Activity				Antifungal Activity			Auxin Producing	Morphology					
	Protein	Cellulose	Lignin	Lipid	*P. capsici*	*R. solani*	*B. cinerea*		Color	Texture	Shape	Elevation	Margin	Color
CGM03	+++ *	+++	O		O		O		Cream	Mucoid	Irregular	Flat	Undulate	Cream
CGM05								O	Beige	Smooth	Round	Convex	Undulate	Beige
CGM07	+++	+++		O	O	O	O		Cream	Mucoid	Irregular	Flat	Undulate	Cream
CGM09	+++	+++	O	O				O	Brown	Smooth	Irregular	Flat	Curled	Brown
CGM11	+++	+++	O				O		Cream	Mucoid	Irregular	Flat	Undulate	Cream
CGM12	+++	+++	O				O		Cream	Mucoid	Irregular	Flat	Undulate	Cream

* Diameter of transparent zone: +, 1~5 mm; ++, 6~10 mm; +++, >11 mm.

## Data Availability

All data are available from the corresponding author upon request.
